# Open reduction and compression with double Kirschner wires for the treatment of old bony mallet finger

**DOI:** 10.1186/s13018-019-1513-2

**Published:** 2019-12-21

**Authors:** Junjun Tang, Kejian Wu, Jinchang Wang, Jian Zhang

**Affiliations:** Department of Orthopedics, The Forth Medical Center of the General Hospital of People’s Liberation Army of China, Haidian District, Beijing, China

**Keywords:** Mallet finger, Fracture fixation, Avulsion fracture, Flexion angle, Kirschner wires

## Abstract

**Background:**

The management of old bony mallet fingers is complicated. The aim of the study is to present a new method of open reduction and compression with double Kirschner wires (K-wires) in treating old bony mallet fingers.

**Methods:**

This was a retrospective analysis of patients with old closed bony mallet fingers treated between June 2013 and December 2016. Complications were observed. The range of motion (ROM) of the DIP joints was measured using a protractor. At the last follow-up, anteroposterior and lateral X-ray of the affected finger was performed, the flatness of the articular surface was scored, and the results were graded using Crawford’s criteria.

**Results:**

Seventeen patients were followed up for 8 (6–19) months. The width of the avulsion fracture block accounted for 25–62% of the articular surface of the distal phalanx. Twelve (70.6%) patients had anterior dislocation of the interphalangeal joint. All patients reported healing at the fracture sites. Healing time was 7.6 ± 2.1(5–13) weeks. All patients had incision healing of I/Class A. Lateral X-ray showed 13 and four patients had excellent and good articular surface flatness, respectively. At the last follow-up, no traumatic arthritis was present. Only one patient developed mild pain after surgery (VAS score of 3). Postoperative ROM was 76.5 ± 10.6° (*P* = 0.0625 vs. healthy side). At the last follow-up, the angle of loss of dorsiflexion was 0–10° (*P* < 0.0001 vs. baseline). The flexion angle was 50–90° (*P* = 0.0625 vs. healthy side).

**Conclusions:**

Open reduction and compression with double K-wires is feasible in treating old bony mallet finger.

## Background

The bony mallet finger refers to the avulsion fracture at the phalangette basilar part caused by traction of the extensor tendon when the original trauma occurs, leading to a limited extension of the distal phalanx. If the treatment is not appropriate, swan-neck deformity of the finger may occur due to the imbalance of the dorsiflexion muscle strength between the proximal and distal interphalangeal (IP) joints [1–3]. The injury time for mallet finger within 4 weeks is defined as being acute, and old when the injury time exceeds 4 weeks [4].

Acute bony mallet fingers are generally treated by splinting or brace fixation. For old bony mallet fingers caused by improper treatment or treatment failure in the acute phase, if the flexion deformity is > 40° or the dorsiflexion function is limited, surgical treatment is needed [1, 5–7]. Currently, there are many surgical fixation methods for the management of bony mallet finger, and the most commonly used ones include Kirschner wire fixation, tension band fixation, and mini-plate or screw fixation [5].

In 1988, Ishiguro et al. proposed closed reduction and K-wire blocking for fixation when treating mallet fingers with avulsion fractures, which had achieved good efficacy [8, 9]. Currently, it is widely used in clinical practice, and some modified methods have been adopted [10]. The main issue with these methods is that they use a K-wire to compress the fracture block from the dorsal side, which has poor control of the avulsed fracture block, and loss of fracture reduction occurs easily after surgery, affecting the flatness of the joint and finger function. In addition, for old bony mallet fingers, because scar tissue is present in the unreduced fracture spaces, a good reduction is impossible, thus adversely affecting postoperative fracture healing and functional recovery.

Therefore, this study proposes a modified reduction method for old bony mallet fingers using open reduction, one K-wire for fixation of the distal IP (DIP) joint, two K-wires for dorsal compression of the avulsion fracture block, and K-wire distal interlocking fixation. This paper presents the clinical outcomes of 17 patients that received this treatment.

## Methods

### Patients

This was a retrospective analysis of patients with old closed bony mallet fingers treated between June 2013 and December 2016. The study was approved by the research ethics committee of the Forth Medical Center of the General Hospital of People’s Liberation Army of China (2019KY014-HS001). The need for informed consent was waived by the committee.

The inclusion criteria were (1) injury time was > 4 weeks; (2) the lateral film of the finger showed the phalangette basal avulsion fracture, and the fracture block represented ≥ 25% of the articular surface of the distal phalanx; and (3) the affected finger could be passively extended. The exclusion criteria were (1) comminuted fracture, (2) width of the fracture block was < 2 mm, (3) DIP joint stiffness, or (4) malunion.

### Surgical procedures

After local anesthesia using lidocaine, a tourniquet was placed at the root of the affected finger. A dorsal H-shaped incision was made over the DIP joint to expose the fracture. The fractured block was separated, and the extensor tendon was released. The scar tissue was removed using a micro-curette to expose and refresh the fracture surface. In the presence of an anterior dislocation of the interphalangeal joint and in the impossibility of reducing it manipulatively, detachment was used to separate and release the palmar articular capsule from the dorsal side through the DIP joint. A 1-mm double-headed K-wire was inserted from the palmar residual articular surface of the phalangette under maximum flexion of the distal phalanx of the affected finger until the end was completely immersed in the distal phalanx. The distal phalanx of the affected finger was placed in slight dorsiflexion, and the K-wire was inserted back into the middle phalanx to stabilize the DIP joint in a slight overextension position. Pointed tweezers were used to reduce the avulsed bone block and maintain it under mild compression. Two 0.8-mm K-wires were crosswise or parallelly inserted in the middle phalanx from the dorsal side percutaneously; the angle between the K-wires and the middle phalanx was about 30°. The K-wires had to be close to the avulsed bone block when inserting, and the space between the two wires at the bone block had to be about 1 mm. Finally, the distal ends of the two K-wires were bent into a U shape and were fixed by interlocking them with the distal end of the first K-wire. Therefore, at this time, the avulsed bone blocks were compressed and fixed to the fracture end by two K-wires. X-ray photographs were taken to confirm the complete reduction of the fracture and good compression and fixation. Finally, the incision was closed.

Some important points have to be noted. First, a double-tip K-wire should be used for fixing the DIP joint in a retrograde manner. The insertion point must avoid the fracture section, especially for avulsed bone blocks representing > 1/3 of the articular surface. Second, the two compressed K-wires are inserted percutaneously to facilitate their removal after fracture healing. Third, the insertion point of the compressed K-wire is located at a distance of 1/3 of the middle phalanx and needs to be close to the avulsed bone. The two K-wires need to be located on both sides (radial and ulnar sides) of the bone block. Then, the bone block can be compressed uniformly from the two sides by two bent K-wires interlocked with the first K-wire. Fourth, the compression force can be adjusted by shaping the distal ends of the two K-wires, and the skin between the two K-wires should not be squeezed in order to avoid ischemia. Finally, before dressing, the tourniquet should be removed, and the peripheral blood supply should be observed to avoid ischemia of the distal end of the finger caused by the DIP joint being in flexion for a long time.

### Postoperative management

The dressing was changed 48 h after surgery. The skin at the incision and the peripheral blood supply of the finger were observed. No plaster or brace fixation was needed after surgery. The patient needed to exercise the other IP joints, and X-ray reexamination was performed every 2 weeks. At week 6, the K-wire was removed without anesthesia and incision. Twenty-four hours after removing the K-wire, the patient was allowed flexion and extension of the DIP joints.

### Data collection

**Fig. 1:**
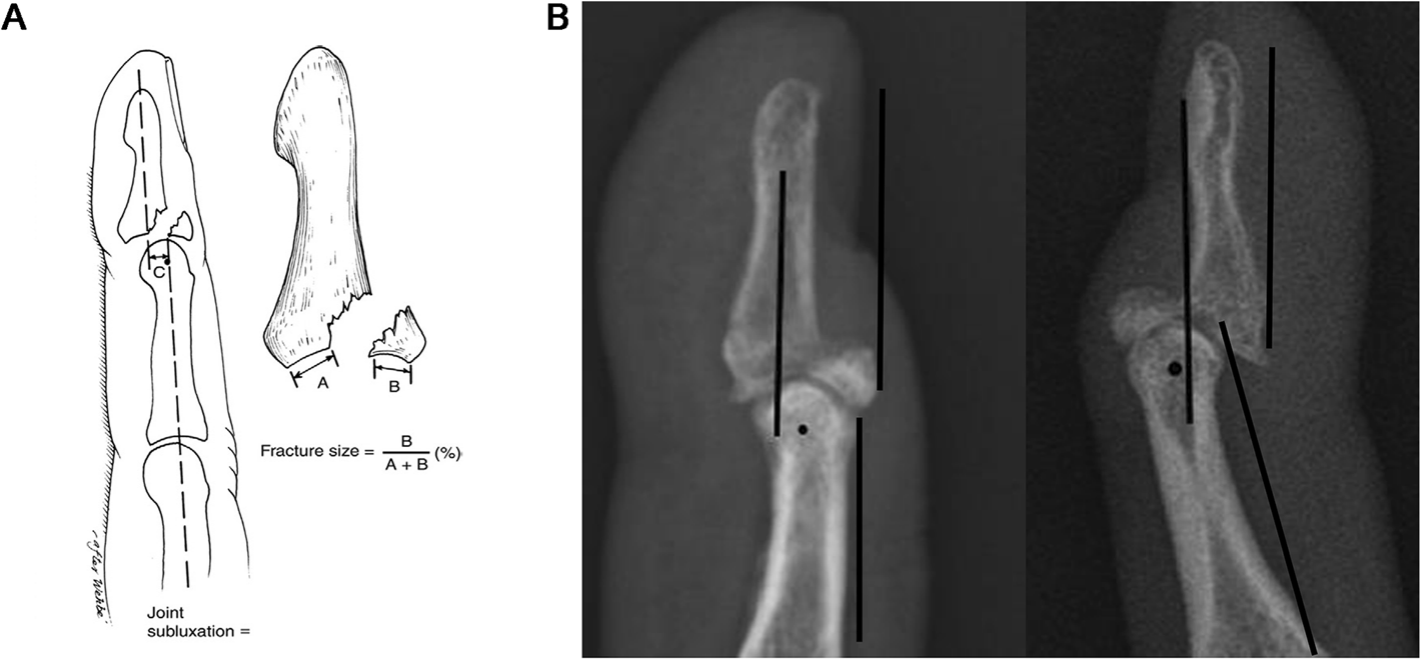
Diagram of the degree of dislocation and the size of the fracture block. **a** The length of the articular surface of the phalangette fracture block and the length of the residual articular surface were measured. The length of the articular surface of the fracture block divided by the sum of the two was the proportion of the articular surface of the fracture block. **b** The lateral film was used to assess whether there was anterior subluxation in the DIP joint of the affected finger. The presence or absence of consistency in the axis of the distal and middle phalanxes was used to determine the dislocation. If the distal phalanx axis was displaced forward, but the dorsal cortical bone line did not exceed the axis of the middle phalanx, it was considered as mild dislocation. If the dorsal cortical bone line was displaced forward and exceeded the axis of middle phalanx, it was considered as severe dislocation

Sex, age, time from injury to operation, affected fingers, and initial treatments were recorded. Using the anteroposterior and lateral films of the finger before surgery, the proportion of the articular surface of the fracture block was measured to assess the size of the fracture block on the lateral film. The lateral film was also used to assess whether there was anterior subluxation in the DIP joint of the affected finger. The presence or absence of consistency in the axis of the distal phalanx and middle phalanx was used to determine the dislocation. If the distal phalanx axis was displaced forward, but the dorsal cortical bone line did not exceed the axis of middle phalanx, it was considered as mild dislocation; if the dorsal cortical bone line was displaced forward and exceeded the axis of the middle phalanx, it was considered as severe dislocation [11, 12] (Fig. 1). The bony mallet finger was classified using the Wehbe and Schneider classification method [3].

### Postoperative follow-up

All patients were followed for the first time 2 weeks after surgery. The complications were observed and recorded. The second follow-up was performed 6 weeks after surgery. The K-wire was removed, and fracture healing was observed. After that, follow-up was performed 4–8 weeks and ended with fracture healing and finger function recovery. At the last follow-up, the time of fracture healing, ROM, VAS score, Crawford score, and articular surface flatness were recorded.

### Assessment indicators

For complications, necrosis of the distal finger, skin necrosis, non-healing or infected wound, and K-wire retraction were mainly observed. A visual analog scale (VAS) was routinely used for pain, 0 point indicated no pain, 1–3 points mild pain, 4–6 points moderate pain, and 7–10 points severe pain. At the last follow-up, the range of motion (ROM) of the DIP joints was measured using a protractor. Taking flexion-extension degree of the corresponding finger on the healthy side as the standard, the degree of loss of dorsiflexion, flexion, and the overall flexion-extension ROM were recorded. The finger function was assessed according to the Crawford criteria [13].

After removing the K-wire, lateral X-ray of the affected finger was performed, and fracture healing was recorded. At the last follow-up, anteroposterior and lateral X-ray of the affected finger was performed, and the flatness of the articular surface was scored: anatomical fracture reduction and no dislocation of the articular surface were considered as being excellent, dislocation of articular surface < 1 mm was considered good, and dislocation of articular surface > 1 mm was considered poor. Meanwhile, the presence or absence of traumatic arthritis was assessed.

### Statistical analysis

Statistical analyses were performed using SPSS 11 (SPSS Inc., Chicago, IL, USA). Continuous variables are presented as medians and quartile (non-normal distribution) according to the results of the Kolmogorov-Smirnov test.

## Results

### Characteristics of the patients

In the present study, 17 patients with an old closed bony mallet finger were included (Table 1). There were 15 males and 2 females; the median age was 24 (17–83) years. There were eight cases of the right hand and nine cases of left hand, including nine ring fingers, four little fingers, three middle fingers, and one index finger. The median time from injury to operation was 54 (30–72) days. Eight patients did not receive preoperative treatment, five received splint fixation, and four had plaster fixation. According to the Wehbe and Schneider classification, there were five type I and 12 type II cases.

**Table 1:** Characteristics of the 17 included patients

No.	Sex	Age (years)	Time from injury to operation (days)	Affected finger	Initial treatment	Classification	Size of the bone block (%)	Degree of dislocation	Time of operation (min)	Post-operative follow-up time (m)	Time of fracture healing (weeks)	Complications	Articular surface	VAS	Crawford score
1	Female	19	35	Right ring finger	No	1A	25	No	64	8	10	No	Good	0	Excellent
2	Male	24	44	Left middle finger	No	2B	33	Mild	66	10	5	No	Excellent	0	Excellent
3	Male	17	58	Left middle finger	Plaster	2B	50	Severe	75	8	6	No	Excellent	0	Excellent
4	Male	24	72	Right ring finger	Brace	1A	28	No	70	12	13	No	Good	0	Excellent
5	Female	27	60	Right little finger	No	2B	40	Mild	48	8	8	No	Excellent	0	Good
6	Male	30	56	Left index finger	Plaster	2B	35	Mild	52	6	8	No	Excellent	0	Excellent
7	Male	31	54	Left little finger	No	1A	28	No	80	9	10	No	Good	0	Excellent
8	Male	18	42	Right ring finger	Plaster	2B	55	Severe	60	9	6	No	Excellent	0	Good
9	Male	19	47	Right little finger	Brace	1B	33	No	50	7	6	No	Excellent	0	Excellent
10	Male	20	66	Left ring finger	Brace	2B	62	Severe	65	8	8	No	Excellent	0	Excellent
11	Male	19	50	Right ring finger	No	2B	52	Severe	55	19	9	No	Excellent	0	Good
12	Male	41	59	Left ring finger	No	2B	40	Mild	52	6	7	No	Excellent	0	Excellent
13	Male	25	60	Left little finger	No	2A	30	Mild	60	6	6	^#^	Good	0	Excellent
14	Male	19	48	Left ring finger	Plaster	2B	50	Severe	54	7	8	No	Excellent	0	Good
15	Male	33	44	Right ring finger	Brace	2B	40	Mild	55	6	5	No	Excellent	0	Excellent
16	Male	22	56	Left ring finger	No	2B	38	Mild	45	8	6	No	Excellent	0	Excellent
17	Male	83	30	Right middle finger	Brace	1B	37	No	58	10	8	No	Excellent	3	Poor
Mean or median		24 (17–83)	54 (30–72)			5/12	2/10/5		58 (45–80)	8 (6–19)	8 (5–13)		13/4		12/4/1

^#^Local black scab

### Preoperative characteristics

The width of the avulsion fracture block in all patients accounted for 25–62% of the articular surface of the distal phalanx, with two cases being < 30%, 10 being 30–50%, and five being > 50%. Twelve (70.6%) patients had anterior dislocation of the IP joint, including seven with mild dislocation and five with severe dislocation.

### Postoperative characteristics

Two weeks after surgery, one patient developed a local black scab at the dorsal skin of the affected finger, which was considered to be from the compression of the K-wire, affecting the blood supply. After the removal of the K-wire 6 weeks, the local scab healed, and there was no long-term complication. In the remaining patients, there was no necrosis of distal fingers, skin necrosis, non-healing or infection, and K-wire retraction.

### Follow-up

**Fig. 2:**
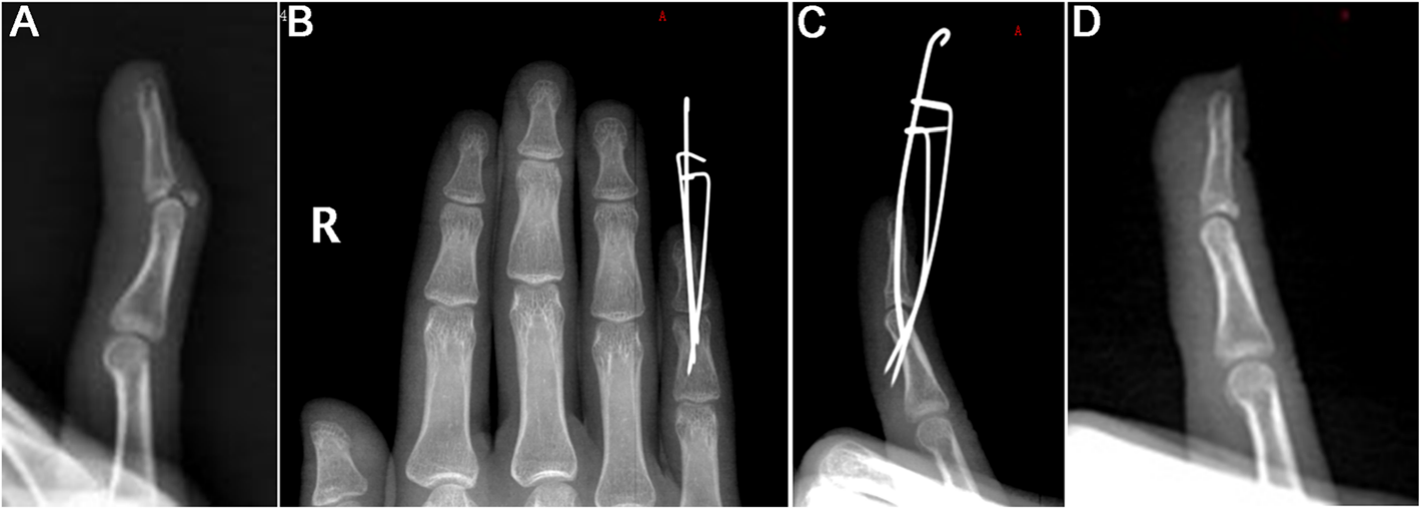
A 27-year-old female patient (no. 5) presented to the hospital 60 days after injury. **a** The old bony mallet finger of the right little finger. **b**, **c** Two K-wires were used to compress and fix the bone block. **d** After removal of the K-wire 6 weeks after surgery, the fracture was initially healed, and the articular surface was smooth without step and collapse

**Fig. 3:**
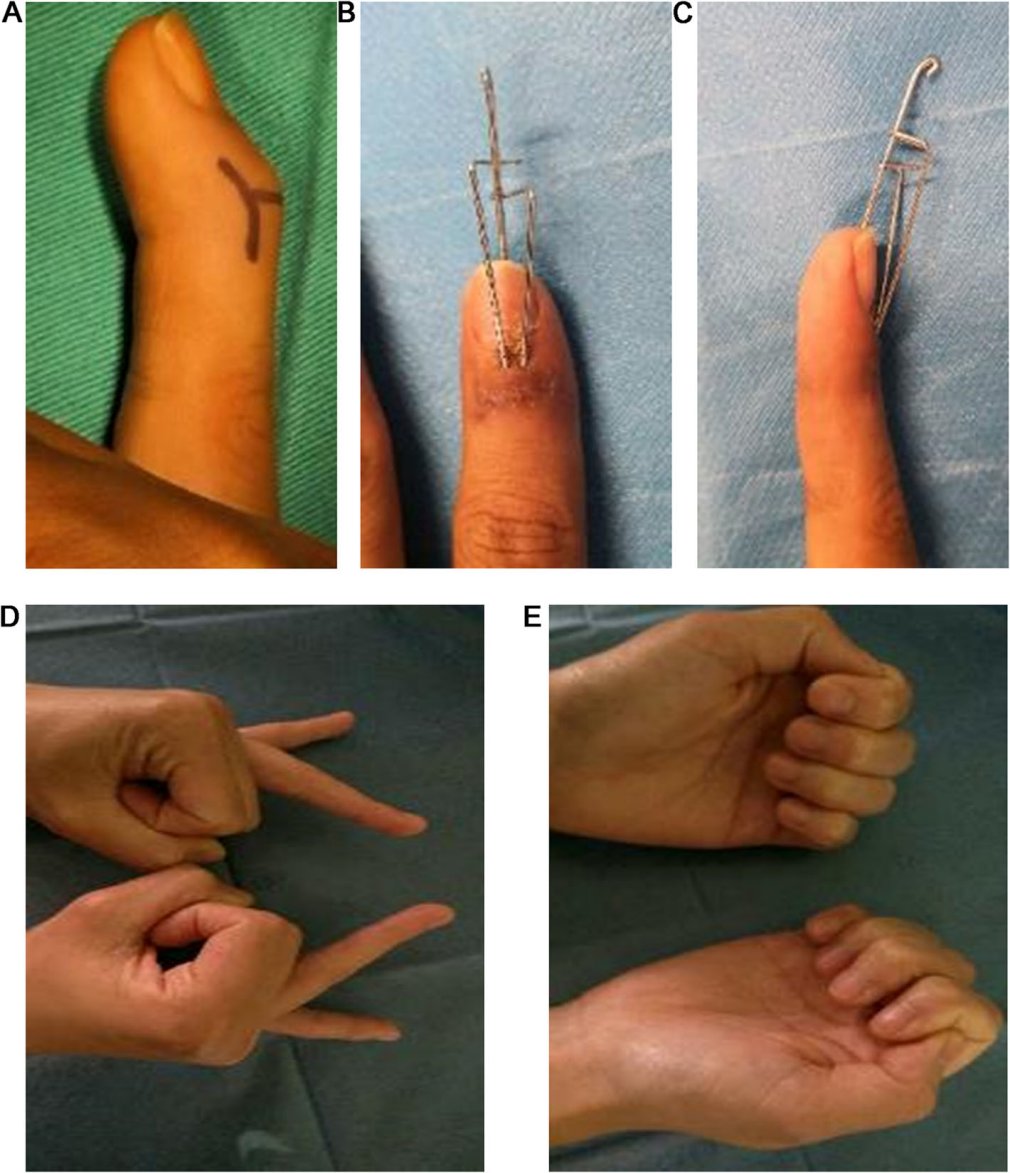
A 27-year-old female patient (no. 5). **a** Preoperative deformity, with limited dorsiflexion. **b**, **c** Anteroposterior and lateral appearance 2 weeks postoperatively. **d**, **e** Appearance 8 months postoperatively. Flexion and extension were normal

Table 2 presents the postoperative data of the 17 patients. All patients were followed for 6–19 months (median of 8 months). All patients reported bony healing at the fracture sites, and the healing time was 5–13 weeks, with an average of 7.6 ± 2.1 weeks. All the patients had incision healing of I/Class A. Lateral X-ray showed that 13 and four patients had excellent and good articular surface flatness, respectively. At the last follow-up, the DIP joints were assessed, and no traumatic arthritis was present. According to the VAS score, only one patient developed mild pain after surgery, which occurred at the flexion and extension of DIP joints; it could be tolerated and painless under resting state. At last follow-up, the angle of loss of dorsiflexion for the DIP joints was 0–10°, with an average of 2.4°, which was statistically different compared with baseline (*P* < 0.0001). The flexion angle was 50–90°, with a median of 80°, which was not statistically different from that of the healthy side (*P* = 0.0625). The postoperative ROM was 76.5 ± 10.6°, which had no statistical difference compared with that of the healthy side (*P* = 0.0625). According to the Crawford assessment standard, in the present study, outcomes were excellent in 12 patients, good in four, and poor in one. Figures 2 and 3 present a typical case.

**Table 2:** Postoperative characteristics of the 17 patients

No.	Angle of loss of dorsiflexion (preoperatively)	Angle of loss of dorsiflexion (postoperatively)	Flexion angle (healthy side)	Flexion angle (postoperatively)	ROM (healthy side)	ROM (affected side postoperatively)
1	35	0	85	85	85	85
2	45	5	55	55	55	50
3	50	0	85	85	85	85
4	30	0	75	75	75	75
5	35	5	80	75	80	70
6	40	0	90	90	90	90
7	40	0	80	80	80	80
8	30	0	75	75	75	75
9	35	0	70	65	65	65
10	40	0	80	80	80	80
11	45	0	90	90	90	90
12	30	0	80	80	80	80
13	25	0	85	85	85	85
14	40	0	80	80	80	80
15	60	10	80	70	80	60
16	45	0	80	75	80	75
17	30	0	85	80	80	75
Mean ± SD						
Median (range)	40 (25–60)	5 (0–10)*	80 (55–90)	80^#^ (50–90)	80 (55–90)	80 (50–90)^&^

*ROM* range of motion

*Comparison between the preoperative angle of loss and postoperative one, *P* < 0.001

^#^Comparison between the flexion angle of affected side and that of healthy side after surgery, *P* = 0.0625

^&^Comparison between the ROM of the affected side and that of healthy side after surgery, *P* = 0.0625

## Discussion

The management of old bony mallet fingers is complicated, and the methods used for acute BMF have poor effectiveness [5, 8–10]. Therefore, the present study aimed to present a new method of open reduction and compression with double K-wires in treating old bony mallet fingers. The results suggest that open reduction and compression with double K-wires may have good clinical outcomes in treating old bony mallet finger.

At present, there are many treatments for acute bony mallet finger, but the optimal treatment method remains controversial [5]. Given that the complications related to skin and soft tissues easily occur during open reduction, for bony mallet finger fractures that involve less than 1/3 of the articular surface, some authors are inclined to suggest conservative treatment, which has achieved good efficacy [3, 14]. If the displacement of the bone mass is obvious, and the joint surface is uneven, especially the size of the bone mass is more than 1/3, many surgeons believe that surgical fixation is needed. The main techniques include closed reduction and dorsiflexion blocking with K-wire or percutaneous screw fixation, as well as open reduction, mini-plate fixation, K-wire fixation, wire drawing technique, and the elastic compression of umbrella handle with the K-wire, all of which achieve good results [5, 14–17]. Nevertheless, in patients with failure of conservative treatment, or those with old bony mallet fingers with injury time > 4 weeks, it is often difficult to reduce the fracture by closed way. Asano et al. [18] treated 10 patients with old bony mallet finger with closed reduction and dorsiflexion blocking with K-wire, and the overall efficacy was satisfactory, but there were problems such as malreduction and articular surface steps. Therefore, for old bony mallet finger, many authors believe that open reduction is needed [19, 20].

For old fracture, the callus and fibrous tissue between the fracture ends will hinder the reduction and affect healing. Therefore, the removal of fracture end and restoring fresh bone interface are necessary for open reduction of old fractures, which is also applicable to old bony mallet fingers. Ishiguro et al. [21] believe that for old bony mallet fingers above 3–5 weeks, the fresh state of the fracture section must be restored. The use of a needle or a K-wire to insert the fracture end to remove the fibrous tissue can partially play a role, but it is impossible to remove the fracture end completely. Reddy et al. [22] used this method to treat the fracture section when treating children with a bony mallet finger, some patients reported insufficient fracture reduction, and the articular surface fracture in the later period was > 1 mm. Therefore, for old bony mallet finger, Reddy et al. suggested that open reduction could achieve better outcomes, by not only removing the fracture ends, but also releasing the adhesion of the extensor tendon to the surrounding tissues. In this way, anatomical reduction of the fracture could be achieved, and steps or collapse of the articular surface after surgery can be avoided.

For bony mallet finger with injury time > 4 weeks, due to the incomplete basal articular surface of the distal phalanx, subluxation to the palmar side is prone to occur because of the continuous traction of the flexor tendon, especially when the avulsed bone block is large. The study by Zhang et al. [23] showed that in patients with bony mallet finger, the incidence of subluxation of the DIP joint could be as high as 46%. In the study by Husain et al. [11], when the fracture block was > 43% of the phalangette articular surface, there was a high probability of subluxation of the DIP joint. This study also revealed that the greater the avulsion fracture block, the higher the incidence of subluxation of the DIP joint, and the more severe the degree of dislocation. If there is a subluxation of the DIP joint, a good reduction and fixation must be performed. Asano et al. [18] believe that anatomical reduction is crucial, and residual subluxation could lead to secondary osteoarthritis. Although the study by Yoon et al. [12] showed that there was no significant difference in efficacy between conservative treatment and dorsiflexion blocking with K-wire when treating bony mallet finger with subluxation of the DIP joint, the enrolled patients were with smaller fracture block and relatively slight palmar subluxation. For old bony mallet finger and subluxation with long injury time, due to palmar contracture caused by long-term flexion state of the DIP joint, closed reduction was unsuccessful in most cases. In the present study, only patients in old fractures were included; when treatment was performed by open reduction, subluxation was difficult to reduce manually, but nerve detachment could be used to release the anterior articular capsule from the dorsal side through IP joint under the traction state. If necessary, a sharp knife blade could be used for partial incision of the articular capsule, thereby achieving a satisfactory reduction. We consider that it is also necessary to accurately reduce the bone mass as much as possible to avoid extension of the extensor tendon, which may lead to a limited dorsiflexion of the distal phalanx. Indeed, a 0.5-mm distension of the extensor tendon lead to a 10° extension deficit, and a 2-mm distension can cause a 40° extension deficit [20].

There are many fixation methods for bony mallet finger, but percutaneous K-wire compression and fixation have been widely used in clinical practice because of small trauma, low cost, and convenient postoperative removal. Nevertheless, to date, most authors use a K-wire to compress the bone block [12, 18]. For acute bony mallet finger, due to the good matching of the fracture ends, a single K-wire for compression after fracture reduction can provide sufficient stability. But for old bony mallet fingers, the fracture ends are often not completely matching, and a single K-wire cannot control the rotation or lateral displacement of the bone block when it is compressed from the middle side. Therefore, modified compression with the double K-wires was performed, and the two K-wires were used to compress from both sides of the bone block. No patient reported displacement of the fracture block after surgery, which greatly improved the stability after the reduction of the bone block.

The K-wire for the fixation of the IP joints after surgery remains a good approach. A fluoroscopic machine is usually needed when inserting the wire during closed reduction. When the avulsed bone block is large, it is difficult to prevent the K-wire from inserting into the fracture end when inserting from the distal end to the proximal end, which will affect the anatomical reduction of the fracture block. For a good position of the K-wire, operations under fluoroscopy not only increase the radiation exposure but also damage the cartilage surface of the IP joint. Moreover, the risk of osteoarthritis will be increased in the later stage. For open reduction, the distal finger can be flexed extremely and pulled to the distal end, and at this time, the residual articular surface of the phalanx base can be exposed. The insertion point is positioned on the articular surface; then, the wire is inserted back and forth through the double-headed K-wire, which can ensure that the K-wire does not involve the fracture end, thus avoiding blocking the fracture reduction. In addition, when closed reduction and blocking with K-wire are used to treat bony mallet fingers, the K-wire is inserted when the distal phalanx is extremely flexed. Therefore, dorsiflexion and fixation of the IP joint cannot achieve complete returning, resulting in an average of 5° of dorsiflexion loss for most patients [18, 23], but the technique in the present study can completely avoid such complications.

At present, some authors [24, 25] advocate closed reduction because complications related to skin and soft tissues easily occur during or after open reduction. On the other hand, the present study suggests that when the procedures are gently performed, the occurrence of such complications was extremely low. There was only one patient with avascular necrosis of partial tissues caused by K-wire compression, and there were no residual complications over the long term. After evaluation, it was determined that the avascular necrosis was caused by compression from the K-wire rather than from the open reduction. With the learning curve and after improving the insertion angle and point, there were no complications related to skin and soft tissues. Nevertheless, because the bone at the distal part is so small, this technique may be suitable for expert surgeons.

At present, there is no uniform conclusion on the time definition of the old mallet finger, but many studies still define an old mallet finger as being older than 4 weeks [1, 4, 18, 22]. At this time, soft tissue due to the fracture gap may block the reduction of the fracture. On the other hand, some authors also used 5 weeks and 3 months as inclusion criteria [16, 20]. Because the main purpose of this manuscript was to introduce the value of this method for fracture reduction and fixation in the treatment of old bony mallet finger, the most commonly used 4-week standard was still adopted for inclusion, but it would be worthy of examining other time points.

In the present study, no night splint was used. Since fracture healing was observed by X-ray at 6 weeks after the removal of the Kirschner wire, and since the distal interphalangeal joint would be stiff for some time due to the long duration of fixation in a slight back extension position, it is probably difficult to lose the reduction. Therefore, there was no use of night support, but it may be indicated in some selected patients.

The main limitation of this study was the lack of a comparison group. In addition, the patients were treated by a single surgeon at a single institution, limiting the sample size and the generalizability of the results. Importantly, there was no comparison group. Much of the historical controls come from Stern et al. [24], the outcomes are often not very different in the absence of instability, and the operation itself is not completely without harm. Prospective randomized controlled trials will be conducted in a later period to confirm the clinical efficacy of this approach further.

## Conclusions

Open reduction and compression with double K-wires for the treatment of old bony mallet finger may be effective and safe. Additional studies are necessary to determine the exact benefits of this approach.

## Data Availability

The datasets used and analyzed during the current study are available from the corresponding author on reasonable request.

## Abbreviations

BMF: Bony mallet finger
DIP: Distal interphalangeal
IP: Interphalangeal
