# Correction to: Open reduction and compression with double Kirschner wires for the treatment of old bony mallet finger

**DOI:** 10.1186/s13018-020-01680-z

**Published:** 2020-04-30

**Authors:** Junjun Tang, Kejian Wu, Jinchang Wang, Jian Zhang

**Affiliations:** Department of Orthopedics, The Fourth Medical Center of the General Hospital of People’s Liberation Army of China, Haidian District, Beijing, China

**Correction to: J Orthop Surg Res (2019) 14: 459**

**https://josr-online.biomedcentral.com/articles/10.1186/s13018-019-1513-2**

Following publication of the original article [1], we have been notified that there was a spelling mistake in the author affiliations. In this Correction, correct and incorrect versions are shown.

Incorrect: Department of Orthopedics, The Forth Medical Center of the General Hospital of People’s Liberation Army of China, Haidian District, Beijing, China.

Correct: Department of Orthopedics, The Fourth Medical Center of the General Hospital of People’s Liberation Army of China, Haidian District, Beijing, China.

## References

[1] Tang J (2019). Open reduction and compression with double Kirschner wires for the treatment of old bony mallet finger. J Orthop Surg Res.

